# Bridging the Gap in Community Care for Patients With Borderline Personality Disorder: Protocol for Qualitative Inquiry Into Patient, Caregiver, and Clinician Perspectives on Service Gaps and Potential Solutions for Severe Emotion Dysregulation

**DOI:** 10.2196/14885

**Published:** 2020-08-20

**Authors:** Laura Friesen, Graham Gaine, Ellen Klaver, Kirsten Klingle, Devashree Parmar, Marianne Hrabok, Jill Kelland, Shireen Surood, Vincent Agyapong

**Affiliations:** 1 Department of Educational Psychology University of Alberta Edmonton, AB Canada; 2 Addictions and Mental Health Alberta Health Services Edmonton, AB Canada; 3 Department of Psychiatry Faculty of Medicine University of Alberta Edmonton, AB Canada; 4 Cumming School of Medicine University of Calgary Calgary, AB Canada

**Keywords:** borderline personality disorder, mental health services, health care quality, access, evaluation, care pathways

## Abstract

**Background:**

Borderline personality disorder (BPD) is characterized by severe emotion dysregulation that is often complicated by comorbid diagnoses, deliberate self-harm, and chronic suicidal ideation. Unfortunately, current care pathways for individuals with BPD are strained by limited resources, inadequate training, and an overuse of emergency departments and crisis teams. Such barriers result in delayed access to effective treatment, which increases risk of deterioration, disability, and morbidity. A first step toward addressing these limitations of the current care pathway is to understand key stakeholders’ lived experiences in this pathway and their perspectives on potential solutions.

**Objective:**

The purpose of this paper is to present a protocol for a study that explores the lived experiences of the current care pathway from the perspectives of patients with BPD, as well as their caregivers and clinicians.

**Methods:**

A qualitative approach is most appropriate for the exploratory nature of the research objective. Accordingly, 3 to 6 patients with a diagnosis of BPD, 3 caregivers of individuals with BPD, and 3 clinicians of patients diagnosed with BPD will be invited to participate in individual, semistructured interviews that focus on service experiences.

**Results:**

It is anticipated that results will yield insight into the lived experiences of patients with BPD, caregivers, and clinicians and provide a better understanding of the perceived gaps in services and potential solutions. Results are expected to be available in 12 months.

**Conclusions:**

This paper describes a protocol for a qualitative study that seeks to understand the lived experiences and perspectives of key stakeholders (patients, caregivers, and clinicians) on the current care pathway for BPD. Results will provide a basis for future research in this area and will have the potential to inform training, practice, and policy.

**International Registered Report Identifier (IRRID):**

DERR1-10.2196/14885

## Introduction

### Borderline Personality Disorder

Severe and pervasive emotion dysregulation is central to borderline personality disorder (BPD). This core trait, along with proclivity for high impulsivity, can increase the risk of engaging in emotionally driven or risky behaviors (eg, unsafe sex, self-harm, problematic relationships) and experiencing adverse events [1,2]. Left untreated, BPD is often accompanied by maladaptive behaviors that can lead to increased risk of suicide and comorbid psychological problems [3-6]. In 2006, an estimated 38% to 73% of individuals with BPD attempted suicide, with approximately 10% of individuals with BPD completing suicide [7]. Now, barely a decade later, estimates of patients with BPD who attempted suicide at least once have increased from 46% to 92% [8]. While rates of suicide are high for this population, not all deaths among individuals diagnosed with BPD are due to suicide. An estimated 14% of patients with BPD die due to causes other than suicide, including cardiovascular disease, substance use, cancer, and accidental reasons [8].

Compounding the risk of suicide is the fact that individuals diagnosed with BPD rarely present with a sole issue or concern. Instead, it is typical for individuals to present with comorbid mental and physical health disorders as well as psychosocial problems [9]. More specifically, individuals diagnosed with BPD can be at a higher risk for depression and anxiety [10], posttraumatic stress disorder, antisocial or criminal behavior [11], homelessness [12], substance use [13], and poor physical health [14]. A further challenge for effective care is that these individuals often present with fluctuating levels of readiness to engage in treatment [15,16]. The complexity of BPD results in high utilization of crisis supports [9] and patterns of inefficient resource utilization [17-19]. Disengagement from services as well as the mismatch between care and patient needs are linked to poorer patient outcomes [20]. The severity of risk, comorbidity, and compounding features associated with BPD have led to a surge of efforts to develop specialized treatments for BPD.

### Treatment for Borderline Personality Disorder

Psychopharmacological treatments for BPD continue to be poorly understood [21], with inconsistent results; therefore, there is no medication that has been officially recognized as effective [22,23]. However, several evidence-based psychological treatments have been developed [24,25]. Arguably the most widely known and empirically tested psychosocial treatment for BPD [24-27] is dialectical behavioral therapy (DBT) [28]; stemming from the cognitive behavioral model, DBT combines cognitive behavioral techniques with principles from eastern philosophy, such as mindfulness to equip individuals with the skills they need to regulate emotions. Another approach, mentalization-based therapy [29,30], draws on psychodynamic principles and attachment theory to help individuals to better understand (ie, mentalize) both their own and others’ thoughts, desires, and intentions and in turn, improve their interpersonal and intrapersonal functioning. Schema-focused therapy [31] integrates cognitive, gestalt, and psychodynamic models of psychotherapy to help identify and change maladaptive frameworks of understanding, or schemas. Evidence suggest that these various approaches to treating BPD result in significant decreases in self-harm, anxiety, depression, and interpersonal problems [32-34].

### Gaps in the Availability of Treatment

While these approaches may be effective in the treatment of BPD, some, like DBT, are resource-intensive and require extensive patient engagement and clinician training. For example, DBT involves weekly individual therapy (typically 1 hour), weekly group skills training (typically 2-2.5 hours), access to telephone coaching of behavioral skills in-between sessions, and weekly therapist consultation team meetings that are designed to support and motivate therapists [28,35]. DBT requires highly trained therapists and many resources to fully operate, which is not always feasible in health care systems that are already facing shortages in staff and resources [36,37]. In turn, wait times for fully adherent DBT programs can range from months to years. Moreover, these programs have eligibility requirements that exclude some individuals with significant barriers to participation, such as low readiness for treatment or significant instability in their social environment.

Due to the extensive resources and training involved in delivering longer-term psychotherapies such as fully adherent DBT, there are alternative approaches used to provide care for individuals with BPD. In particular, many health care systems have DBT-informed groups and shorter-term psychoeducation or skills training groups focused on emotion regulation [38]; however, these services still require patients to be ready for therapy, able to manage treatment interfering behaviors (ie, active self-harm, poor attendance), and participate effectively in group therapy programs. Due to the volatile behaviors and affect that are central to BPD, it is estimated that 46% to 67% of BPD outpatients disengage from these alternative services [39,40]. Therefore, similar to DBT, a substantial number of individuals with the most severe BPD struggle to meet eligibility requirements. Consequently, these individuals tend to become frequent users of crisis teams and emergency departments or get “lost to the system” and otherwise do not receive appropriate care [16,41,42].

Patients with BPD require services that are more accessible, engaging, and stable and that keep them safe and help set goals for their future — regardless of ability or willingness to participate in more structured therapy approaches. One potential solution to this problem is the implementation of stepped care [43-45], a care pathway that matches intervention intensity to patients’ severity and needs [46,47]. A small but growing body of literature suggests that stepped care results in more timely and accessible care and may provide effective treatment for emotion dysregulation [48].

The limited availability of specialized, stepped care undermines the overall effectiveness of health care systems. Perhaps one way to address this problem is to understand the experiences of individuals who have lived experiences in the current care pathway for BPD. The exploratory nature of qualitative research is a viable way to shed light on these experiences. Previous qualitative research demonstrates that patients often feel neglected and stigmatized by health care providers [3,49,50], caregivers feel overlooked by mental health services [50], and providers hold negative attitudes towards individuals with BPD [19,41,51]. While existing data support the frustration experienced by stakeholders, there are no studies that explore these various stakeholder perspectives on the current care pathway in Canada.

### Purpose

The purpose of the present paper is to describe a protocol of a qualitative study that seeks to understand key stakeholders’ (ie, patients with severe BPD, caregivers, and clinicians) experiences with, expectations of, and suggestions for the current care pathway. The protocol is designed to explore two main research questions. First, how do patients with BPD, caregivers, and clinicians experience the health care system during times of severe emotion dysregulation? Second, how do patients with BPD, caregivers, and clinicians think the gaps in care could be addressed?

## Methods

### Design

A qualitative approach and phenomenological method known as interpretative phenomenological analysis (IPA) [52] will be used, wherein researchers use broad and open-ended interviews to explore individuals’ experiences [52,53]. This approach is flexible and non-prescriptive, allowing for the generation of rich information about the experiences of interest [54]. Another method that has frequently been used in interpretive research and that will be employed in this study is the use of pre-interview activities. Pre-interview activities provide prompts for interview topics, aid in memory recall about events, and, therefore, enhance qualitative interviews by holistically exploring a participant’s understanding of their history, context, and the phenomenon of interest [55,56].

### Setting

This study will take place in Edmonton, Alberta under the Addiction and Mental Health program of Alberta Health Services (AHS), the province’s integrated health care system [57,58]. AHS encompass many public health care services, including Emergency Departments, inpatient and outpatient services, and the Family Connections program (which aims to support caregivers of individuals with mental health concerns). Participants will be recruited from a variety of these services.

While there are private psychologists in Edmonton who provide psychotherapy to patients with BPD and could be used as recruitment sites, these services typically charge CAD $200 per hour (the recommended fee by the Psychologists’ Association of Alberta [59]) and are often unaffordable for individuals with severe mental health problems. Moreover, given the high risk of suicidality and high needs common to BPD populations, private psychologists may be likely to refer their patients to emergency departments within the public health care systems or crisis teams when the patient is in acute phases of the illness. Therefore, we will recruit participants from only the public health care system.

### Instrumentation

This study protocol uses pre-interview activities and interview schedules, which are supplemented with audio-recording and field note forms (researcher memos of interviews).

#### Pre-Interview Activities

Pre-interview activities often include lists, diagrams, timelines, drawings, or schedules; this study will use pre-interview activity templates that have been developed by an expert in the field for adaptation to research inquiries [56]. Participants will be sent the pre-interview activity instructions in advance and will be asked to complete at least 4 activities of their choice in the week leading up to the in-person interview. For a full description of the use of the pre-interview activities, the reader is referred to the work by Ellis et al [56].

#### Interview Schedule

The authors of this protocol developed a set of questions through team consultation and based on clinical expertise and methodological literature on the development of IPA interview questions. More specifically, a method called the “funneling technique” guided the development of the interview schedule. This method arranges the topics to be covered from general to more specific or emotionally charged themes [53]. General areas covered in these one-on-one semistructured interviews with participants mirror the research questions about participants’ experiences with, and perceived gaps in, the current care pathway. The interview schedules provide structure and flexibility that allows for the emergence of rich data generation but still maintains a level of consistency across participants [52]. Accordingly, as per the nature of an exploratory study that uses IPA, interviews may not cover all questions and may overlap in the same topic more than once, with all experiences and perspectives being important to the study. The semistructured interview schedules (see Multimedia Appendices 1-3) for each group ensure that the research question(s) are significant and relevant for each individual [53].

### Participants

Patients with BPD, caregivers of patients with BPD, and clinicians of patients with BPD will be invited to participate. Note that it is possible for individuals to present in dyads or triads (eg, patient with BPD, the mother of that patient, and the clinician currently treating that patient); however, this is not expected, simply due to the variability of programs and sites from which participants will be being recruited.

#### Inclusion Criteria

Patients must be current patients at the Edmonton Community Mental Health Clinic, diagnosed with BPD, and aged 18 years or older. Caregivers can be a parent, grandparent, sibling, partner, or close friend and must be a key support in the lives and care of patients diagnosed with BPD and current participants in the Family Connections program [60]. Closeness will be defined as a family, partner, or friend who had direct contact with or key roles in the care of the patient. Clinicians must be mental health therapists, be currently working at Edmonton Community Mental Health Clinic, and work directly with individuals diagnosed with BPD*.*

#### Exclusion Criteria

Patients cannot participate if they are in an active psychosis state, currently suicidal or homicidal, at high risk of acute decompensation, cognitively unable to participate due to drug-related or alcohol-related activities, or determined by Edmonton Community Mental Health Clinic staff as having a cognitive capacity that is too low to participate. Caregivers cannot participate if they are not in close contact or involved in care in some way of an individual who is diagnosed with BPD. Clinicians cannot participate if they are not currently and directly working with or managing cases of individuals with BPD or have less than 1 year of work experience with patients diagnosed with BPD.

#### Sample Size

In accordance with IPA research guidelines [52], this protocol is designed to include a total of 9 to 12 participants who will be recruited through purposive sampling [61]. Of these 9 to 12 participants, between 3 and 6 current patients with a diagnosis of BPD, 3 caregivers of individuals with BPD, and 3 clinicians working with this population will be invited to participate. The variability in the number of participants in the patient group accounts for the variability in how BPD can present, as suggested by the Health Research Ethics Board, University of Alberta.

### Procedure

#### Recruitment

The research team will communicate with representatives and key decision-makers of the public health care sites mentioned and inform clinicians about the study. Clinicians may be invited as potential participants and/or clinicians may have a role in recruiting potential patient participants. Clinicians who agree to partake in the recruitment phase will be informed of inclusion and exclusion criteria to consider when recruiting potential participants. Inclusion criteria will also be stated explicitly in the recruitment posters, recruitment emails, letters of information, and consent forms.

For patients, recruiters from the Edmonton Community Mental Health Clinic will approach individuals who fit the inclusion criteria. The recruiter will inform patients of the study and will provide the patient with a recruitment poster that directs patients to contact the research team for more information. The recruiter will clearly state that patients’ participation in the study will not, in any way, impact their therapy or mental health services, regardless of whether they choose to participate. Recruiters will not be made aware of which patients have chosen to participate in the study.

For caregivers, recruiters from the Family Connections program in Edmonton will introduce the study to caregivers who are attending the program’s group sessions. At the beginning and end of group sessions, recruiters will inform caregivers of the study and pass out copies of the recruitment poster that directs caregivers to contact the research team for more information. The recruiter will clearly state that caregivers’ participation in the study will not, in any way, impact their therapy or mental health services, regardless of whether they choose to participate. Recruiters will not be made aware of which caregivers have chosen to participate in the study.

For clinicians, researchers will use a list-serve email, recruitment posters, and word of mouth to recruit clinicians from the Edmonton Mental Health Clinic. Clinicians will be informed that they are permitted to participate in the interview during work hours and do not need to notify nor request permission to participate from their managers. Clinicians who are interested in participating are directed to use the provided contact information to contact the research team.

### Data Collection

#### Pre-Interview Activities

Approximately one week prior to the interview, the researchers will mail a letter of information, along with a number of pre-interview activities, to participants. Once participants have read the letter of information and confirm to the researcher over phone or email that they are interested in completing the study, the researcher will ask participants to complete the pre-interview activities in the week prior to the interview date. Note that informed consent (written and oral) for official participation in the study happens on the interview date. Pre-interview activity data will not be collected prior to informed consent. Just as with the interview schedules, pre-interview activities are adapted to each participant group, to explore their perspectives. Participants will be asked to complete at least 4 pre-interview activities of their own choice from the options provided; pre-interview activities generally take from 5 to 30 minutes each to complete but participants can take as much time as they wish on these. Participants will be asked to bring the completed pre-interview activities to the interview to discuss prior to the open-ended questions.

#### Interview Schedules

Interviews will be conducted by one of our research team members (LF) who is trained in qualitative inquiry research, has experience using IPA in exploratory studies, and has published original IPA research findings. Upon meeting the participant, the interviewer will carefully review the information and consent form with each participant (see Ethical Considerations for details on informed consent process), which includes informing the participant of the presence of the audio-recording and the procedures used to protect their anonymity. Participants who agree to participate will be asked to sign the information and consent form.

The interview portion of the study will take 1 to 1.5 hours, starting with a discussion of completed pre-interview activities. Interviews will be conducted in person to monitor participant reactions and to effectively use the pre-interview activities (the interviewer needs to be able to see the completed pre-interview activities). Interviews will occur at one of three AHS community clinic locations for participant convenience.

### Data Analysis

Expected data analysis will be guided by the step-by-step guide to conducting IPA research by Smith et al [52], which involves 6 steps.

#### Reading and Re-Reading

Researchers become familiar with the transcribed interviews by reading and re-reading the transcripts several times.

#### Coding

The researcher begins to consider possible themes that seem to be emerging; this method is called “preliminary exploratory analysis” [62]. Researchers will code the data in sections of text.

#### Clustering

Higher levels of abstraction will occur when codes are grouped into common themes; this method has been called “clustering” [53]. The researcher codes data into themes and subthemes while continuously seeing the data as a whole [52].

#### Iteration

Researchers begin an iterative process that may have many revisions during which the meaning of participants’ small stories fit into the context within the narrative [52]. Researchers constantly check back to ensure that the themes cohere to the raw transcription data. The themes will be organized in an analysis table to track the themes, subthemes, and direct quotes from transcriptions that support the themes.

#### Narration

The researcher will develop a narrative of the findings, describing relevant themes and using direct quotations to support the interpretations that have been made.

#### Contextualization

Researchers will further interpret the findings in view of existing literature on the topic.

### Ethical Considerations

The current study has received ethical clearance from the University of Alberta’s Health Ethics Research Board (Ref. # Pro00086416). The regional health authority, AHS, has provided operational approval for the research sites (AHS #45583; #45585) and Edmonton Zone Administrative Approval (AHS #36068). Issues surrounding privacy, confidentiality, and data retention that are particularly pertinent to the present protocol are discussed in the next sections.

#### Evaluation of the Research

Given that qualitative research is inherently interpretive, it is understood that the researchers are as much a part of the study — including the data analysis process — as the participants [62]. The interpretive and potentially subjective nature of this research makes it important that interpretation of data and findings are verified by a source outside of the principal researcher to ensure trustworthiness and confidence in the results. Therefore, the present protocol involves member checking and external audits to enhance trustworthiness and confidence. Member checking requires the researcher to check with participants regarding the preliminary findings by asking clarifying questions used to enhance and deepen the interpretations, as they arise in the interview with the participant. External auditing considers the opinions of another person such as a co-researcher or research supervisor who reviews the codes and themes in search of continuity and validity [62]. The research findings will also be thoroughly and carefully evaluated by the research team for coherence, persuasiveness, comprehension, and usefulness [63,64].

#### Voluntary Participation

All participants will be informed that participation is completely voluntary. Even after participants have provided informed consent to participate in the pre-interview activities and interviews, participants will be free to exit or end their participation in the interview at any time. Participants will be free to ask to withdraw any of their comments. Participants will be given a 4-week time frame after their individual interviews to withdraw their data. These rights are outlined in the information letter and consent form, and participants will be reminded of these rights when they complete the informed consent form and at the beginning and end of the interview.

#### Privacy of Information

Only the research team will have access to research data. All identifying information will be removed from any materials produced by participants (ie, pre-interview activities and transcribed interviews). All participants must provide informed consent to use the pre-interview activities in the research process, including publication. Images of pre-interview activities will not be disseminated without the consent of the participants. Identifying information in pre-interview activities, if present, will be “blacked out.” If this is not possible, the pre-interview activity image itself will not be published but will only be described in research documents. Finally, the participant’s name will not be attached to the pre-interview activities, and a pseudonym or number will be used when describing the conversation that comes from the material created. After the completion of the study, participants’ information (eg, name, phone number, and email) will be destroyed.

#### Confidentiality

The list of the participant names and their respective contact numbers will be confidential and securely stored separately from the data. The data and participant names will be kept in a locked filing cabinet in the research office and will only be available to the research team. All computer files (eg, transcriptions of the data) will be kept in a password-protected file that will only be accessed by the research team. Files will be kept on the secure AHS shared drive. Audio-recorders equipped with USBs will be used and will be stored in a secure location. Recordings will be deleted off the audio recorders after the audio file has been transferred to a secure research file on the researchers’ secure AHS computer. Any internal communication between team members is done in-person or within the secured AHS network.

#### Record Retention

All data pertaining to the study will be kept for a minimum of 5 years and will be securely stored in a locked filing cabinet in the secure research office. All computer files (eg, transcriptions of the data, analysis documents, memos) will be kept in a password-protected file that will be stored on the secure AHS network.

## Results

Using the presented protocol, it is expected that themes will emerge for each participant, then within the groups (patient, caregiver, and clinician), and finally, across all groups. The main goal of this qualitative analysis is to allow for the emergence of themes that illuminate the perspectives on the current care pathway for patients with BPD, perceived gaps, and potential solutions. Recruitment will commence February 2019, and the findings of the study are expected to be available in 12 months following the publication of this protocol. The findings may be used for the development or modifications of programs for patients of AHS. In addition, articles will be written for peer-reviewed journals and dissemination at conferences.

IPA does not predetermine hypotheses but instead, all and any results are welcome. Based on existing theoretical knowledge and clinical experiences, it is foreseeable that patients with BPD will be able to describe the strengths and weaknesses of the current care pathway and will provide the most critical insights into needs and potential service solutions. It is also foreseeable that caregivers will be able to provide important information on how natural supports are challenged by shortcomings in the system and how to address those gaps. It is predicted that clinicians are aware of the existing gaps in services and clinical training and will have valuable and practical solutions to overcoming these gaps.

## Discussion

### Overview

The present paper describes a protocol for a qualitative study that seeks to understand the experiences, perceived gaps in services, and potential solutions in health care services for individuals with BPD from these 3 stakeholder groups: patients with BPD, caregivers, and clinicians. The interviews will have certain commonalities in terms of structure and topics covered across the 3 groups of participants. We hope to learn about the unique aspects of each perspective as well and the practical implications for the health care system.

Patient perspectives are invaluable in a health care system that strives to be patient-centered [58]. It is our hope to better understand, from a patient’s perspective, what it is like to live with BPD and navigate the health care system. For example, we hope to learn more about patients’ interactions with providers in the health care system, their process in accessing and accepting help, factors of care that helped or hindered their recovery, and suggestions on how to improve services for this population from their own perspectives. From caregivers, we hope to learn what it is like to care for someone who is diagnosed with BPD, to learn about the needs of the caregivers in the caring process, and what caregivers think patients with BPD need. This information can help to inform program planning and caregiver involvement in treatment and determine the need for more or different types of support required by caregivers. From clinicians, we hope to understand if, how, when, and why clinicians’ experiences working with this population have changed over time. Accordingly, we hope to learn about what clinicians need to effectively work with this population and about the outcomes they have seen in their treatment of this population. We also hope to understand what the clinicians believe the patients need as well as the strengths that clinicians perceive in this population. This information can help inform processes and procedures within the system.

### Limitations

Provided the small sample size inherent to this methodology, findings are not generalizable in the traditional, quantitative sense. This is a consideration of the current protocol when using the results to determine systemwide changes. Instead, this protocol provides an in-depth analysis of a small population of participants with shared experiences, which can guide future research. Still, consumers of qualitative research can take the findings and apply the new understandings to their own context, leading to helpful action in environments, populations, or scenarios that may differ from the cases presented in the research of interest [65].

### Conclusion

This study will enhance our understanding of an important gap in our current health care system by exploring multiple perspectives on the care pathway for those with BPD. The findings of the study have the potential to inform training, practice, policy, and future research in this area. The aim of this exploratory research is to develop better understandings that can lead to helpful action with this population [56]. Accordingly, results can be used to inform addiction and mental health program planning within Edmonton and to disseminate the learnings to other jurisdictions.

## Appendix

Multimedia Appendix 1 Interview schedule for patients.

Multimedia Appendix 2 Interview schedule for caregivers.

Multimedia Appendix 3 Interview schedule for clinicians.

## Abbreviations

AHS: Alberta Health Services
BPD: borderline personality disorder
DBT: dialectical behavioral therapy
IPA: interpretative phenomenological analysis
