# Supplementary material for: Bridging the Gap in Community Care for Patients With Borderline Personality Disorder: Protocol for Qualitative Inquiry Into Patient, Caregiver, and Clinician Perspectives on Service Gaps and Potential Solutions for Severe Emotion Dysregulation
Source: JMIR Res Protoc. 2020 Aug 20;9(8):e14885. doi: 10.2196/14885 (PMC7471890; doi:10.2196/14885)
Supplement: Multimedia Appendix 1 [file resprot_v9i8e14885_app1.docx]

**_________________________Interview** **Schedule - Patients_________________________**

| Participant Demographics | |
| --- | --- |
| Age: |  |
| Gender: |  |
| Time since diagnosis of BPD: |  |
| Other addiction and mental health diagnoses: |  |
| Medical diagnoses: |  |

**Opening Question:**

What drew you to our study?

**[Discussion of completed Pre-Interview Activities (PIAs)]**

**Semi-Structured Questions:**

1. Tell me what it has been like to have BPD?
2. What did you expect from the healthcare system?
   1. Probe: What did you expect for treatment of BPD?
3. Tell me about your contact with the healthcare system?
   1. Probe: Tell me about your contact with the healthcare system when you are struggling [use their language].
4. How did the healthcare system do well?
   1. Probe: How did it support you when you were [struggling]?
5. How could the healthcare system have done better?
   1. How could the healthcare system have done better when you were [struggling]?
   2. What barriers came up, if any?
6. What were your needs along the way?
   1. What were your needs when you were [struggling]?
   2. What were your caregivers’ needs along the way?
7. What would you like to see in the future from the healthcare system?
   1. What would have made this a better experience for you?
   2. …when you were [struggling]?

**Closing Question:**

You discussed what drew you to our study at the beginning. I’m sure you had some expectations coming in (like what we would ask you/what you wanted to talk about). Is there anything you thought we would ask that we didn’t or anything you wanted to discuss that we didn’t cover?

***[General debrief following interview: go over main points in letter of information, check in on emotional status to ensure safety after potentially sensitive conversation. Provide referral resources if necessary]
