# Supplementary material for: Bridging the Gap in Community Care for Patients With Borderline Personality Disorder: Protocol for Qualitative Inquiry Into Patient, Caregiver, and Clinician Perspectives on Service Gaps and Potential Solutions for Severe Emotion Dysregulation
Source: JMIR Res Protoc. 2020 Aug 20;9(8):e14885. doi: 10.2196/14885 (PMC7471890; doi:10.2196/14885)
Supplement: Multimedia Appendix 2 [file resprot_v9i8e14885_app2.docx]

**_________________________Interview** **Schedule - Caregivers_______________________**

| Participant Demographics | |
| --- | --- |
| Age: |  |
| Gender: |  |
| Relationship to Patient: |  |
| Time since diagnosis of relative’s BPD: |  |
| Other addition and mental health diagnoses of relative: |  |
| Medical diagnoses of relative: |  |

**Opening Question:**

What drew you to our study?

**[Discussion of completed Pre-Interview Activities (PIAs)]**

**Semi-Structured Questions:**

1. Tell me about what it has been like to have a relative with BPD?
   1. How has it impacted your life?
2. What did you expect from the healthcare system?
   1. What did you expect for treatment of BPD?
3. Tell me about your relative’s contact with the healthcare system?
   1. Can you tell me about their contact with the system when they were [struggling – *use participants’ language*]?
4. Tell me about your contact with healthcare system for your relative’s treatment?
5. What did the healthcare system do well?
   1. … when they were [struggling]?
6. How could the healthcare system have done better?
   1. … when they were [struggling]?
   2. What barriers came up, if any?
7. What were your relative’s needs along the way?
   1. … when they were [struggling]?
8. What were your needs along the way?
   1. … when your relative was [struggling]?
9. What would you like to see in the future from the healthcare system?
   1. What would have made this a better experience for you?
   2. … when your relative is [struggling]?

**Closing Question:**

You discussed what drew you to our study at the beginning. I’m sure you had some expectations coming in (like what we would ask you/what you wanted to talk about). Is there anything you thought we would ask that we didn’t or anything you wanted to discuss that we didn’t cover?

*[General debrief following interview: go over main points in letter of information, check in on emotional status to ensure safety after potentially sensitive conversation. Provide referral resources if necessary]
