# Supplementary material for: Bridging the Gap in Community Care for Patients With Borderline Personality Disorder: Protocol for Qualitative Inquiry Into Patient, Caregiver, and Clinician Perspectives on Service Gaps and Potential Solutions for Severe Emotion Dysregulation
Source: JMIR Res Protoc. 2020 Aug 20;9(8):e14885. doi: 10.2196/14885 (PMC7471890; doi:10.2196/14885)
Supplement: Multimedia Appendix 3 [file resprot_v9i8e14885_app3.docx]

**_________________________Interview** **Schedule - Clinicians_______________________**

| Participant Demographics | |
| --- | --- |
| Age: |  |
| Gender: |  |
| Healthcare Profession: |  |

**Opening Question:**

What drew you to our study?

**[Discussion of completed Pre-Interview Activities (PIAs)]**

**Semi-Structured Questions:**

1. Tell me about what it has been like working with this population?
   1. What has it been like when they are [struggling – *use participant’s language*]?
2. What did you expect it would be like to work with this population?
3. What does the healthcare system do well with this population?
   1. What do you find is effective?
   2. …when they are [struggling]?
4. How could the healthcare system do better?
   1. … when they were [struggling]?
   2. What barriers or challenges came up, if any?
5. What are the needs of this population along the way?
   1. … when they are [struggling]?
6. What strengths have you noticed in this population?
7. What do clinicians need to work effectively with this population?
   1. What training is needed?
   2. What supports are needed?
   3. Did you get what you needed?
8. What outcomes have you seen when working with this population?
9. Ideally, how would you think the healthcare system could best serve this population?
   1. What would have made this a better experience for your client when they were [struggling]?
   2. What new programs/services are needed?

**Closing Question:**

You discussed what drew you to our study at the beginning. I’m sure you had some expectations coming in (like what we would ask you/what you wanted to talk about). Is there anything you thought we would ask that we didn’t or anything you wanted to discuss that we didn’t cover?

***[General debrief following interview: go over main points in letter of information, check in on emotional status after potentially sensitive conversation. Provide referral resources if necessary]
